# Editorial: It takes a village: The expanding multi-disciplinary approach to brain metastasis

**DOI:** 10.3389/fonc.2022.1054490

**Published:** 2022-10-19

**Authors:** Peter E. Fecci, Ganesh Rao, Priscilla K. Brastianos, Gavin P. Dunn, Carey K. Anders

**Affiliations:** ^1^ Department of Neurosurgery, Duke University School of Medicine, Durham, NC, United States; ^2^ Duke University School of Medicine, Duke Center for Brain and Spine Metastasis, Durham, NC, United States; ^3^ Department of Neurosurgery, Baylor College of Medicine, Houston, TX, United States; ^4^ Central Nervous System Metastasis Program, Massachusetts General Hospital, Boston, MA, United States; ^5^ Department of Neurosurgery, Massachusetts General Hospital, Boston, MA, United States; ^6^ Department of Medicine, Division of Medical Oncology, Duke University School of Medicine, Durham, NC, United States

**Keywords:** brain metastasis, Leptomeningeal disease, melanoma, lung cancer, breast cancer, multi-disciplinary approach

## Perspective

Brain metastases (BrM) represent the most common adult intracranial malignancy and continue to herald relatively poor survival. Approximately 180,000 to 216,000 of the 1.44 million cancer patients in the United States will develop BrM each year (1, 2). Ultimately, 20 to 40% of patients with solid cancers will develop BrM over the course of their advanced disease (1, 3). The risk of BrM varies considerably between primary cancer types, although the most common sources consist of lung (50-60%), breast (15-20%), and melanoma (5-10%), followed by renal cell, colorectal, pancreas, and urologic/gynecologic cancers (4–6). This incidence is approximately 20-fold higher than glioblastoma, the most common primary brain cancer, and nearly 3-fold higher than the incidence of all primary brain tumors combined (7, 8). The prevalence of BrM has continued to increase as improvements in cancer screening methods and extracranial systemic treatments, including immunotherapy, have evolved. Thus, patients are increasingly surviving longer such that later disease sequelae, including intracranial progression, are more common occurrences (9, 10). As a result, BrM have now evolved into a leading cause of both morbidity and mortality across many types of advanced cancer.

Despite the increasing demand, few BrM-specific therapies exist, and integrated programmatic, collaborative approaches toward BrM research have been virtually absent. Multi-disciplinary efforts to devise and discern novel therapies are desperately needed. Such efforts will require the inputs of key contributing subspecialties, which include (but are not limited to) medical or pediatric oncology, neuro-oncology, neurosurgery, radiation oncology, neuroimaging, neuropathology, and palliative care. The last few years has seen the emergence of coordinated “brain metastasis clinics” at a handful of medical centers, offering patients access to varying modes of multi-disciplinary care, BrM-focused clinical trials, and more advanced treatment recommendations from cooperative tumor boards. For true progress in clinical care to be made in the imminent future, however, these team-based approaches to clinical care will need to evolve from exception to norm.

Akin to what has been seen with clinical care, much of the research performed to date on BrM has been performed in silos, focusing on a single disease histology (i.e., melanoma), or a single phase or facet of tumor progression (i.e., immune evasion, microenvironment, or tumor cell signaling). As a result, there has been a failure to capitalize on successes or knowledge gains that can: 1) inform across disease groups; 2) link scientific approaches, such as genomics, immunology, and cell signaling; 3) overcome central nervous system (CNS)-imposed treatment barriers; and 4) determine overlapping networks common to brain metastatic progression in disease-agnostic fashion. Furthermore, breaking down silos in the research arena can at times be more challenging than doing so in the clinical realm, as recognizing and incentivizing fruitful research collaboration can provide unique challenges. Granting agencies have yet to foster team science aimed at BrM to the extent that they have for primary cancers or brain tumors, an important short-coming when one considers the greater dependence on multi-disciplinary therapeutic approaches that characterizes BrM. Research “requests for applications” (RFAs) aimed at identifying and bringing together scientists whose work may be wittingly or unwittingly applicable to BrM must be brought forward, and multidisciplinary conferences focused on the same likewise further emerge. The recent development of a focused annual meeting on brain metastasis by the Society of Neuro-oncology and the American Society of Clinical Oncology represents a notable victory for recognition and highlights an appropriate future direction for the field.

In the same vein, this collection of articles (see below summary) represents the editors’ efforts to call attention to the key quandaries facing those tackling BrM from both a clinical and research perspective, as well as to consolidate those quandaries into one approachable resource. Identifying, agreeing upon, and properly focusing attention on these issues will be an important first step for the field. While historical questions have been of the genre of i.e. increasing drug access across the blood-brain barrier (BBB), newer questions will perhaps focus instead on molecular differences across primary tumor and metastasis, personalized approaches, CNS-specific immune evasion, and mechanisms of leptomeningeal spread, to name a few. The goal of this issue is to stimulate discourse, foster collaboration, and shed light on a growing population of patients whose needs are currently outstripping our provided options.

## Summary of articles

A number of articles in this collection offer varying and thoughtful angles on the modern evolving approaches toward the diagnosis, management, and study of BrM (Brenner and Patel, Kirkpatrick, Ene and Ferguson, Sarmey et al). Others aim to interpret recent clinical trial results (Tan et al, Taslimi et al); tackle brain-specific concerns within the tumor microenvironment (Toh et al, Heet et al); or highlight the increasing use of detailed molecular and immunogenomic profiling for the purpose of creating personalized and targeted therapeutics (Shen et al, Routh et al). Finally, as our capacity grows for improving survival amongst patients with BrM, so must we develop new focus on issues of survivorship. We thus also include articles that address delayed treatment effects such as radiation necrosis (Park et al), as well quality of life more broadly (Wu et al).

## Author contributions

All authors listed have made a substantial, direct, and intellectual contribution to the work and approved it for publication.

## Conflict of interest

The authors declare that the research was conducted in the absence of any commercial or financial relationships that could be construed as a potential conflict of interest.

## Publisher’s note

All claims expressed in this article are solely those of the authors and do not necessarily represent those of their affiliated organizations, or those of the publisher, the editors and the reviewers. Any product that may be evaluated in this article, or claim that may be made by its manufacturer, is not guaranteed or endorsed by the publisher.
